# Pegylated-asparaginase during induction therapy for adult acute lymphoblastic leukaemia: toxicity data from the UKALL14 trial

**DOI:** 10.1038/leu.2016.219

**Published:** 2016-09-09

**Authors:** B Patel, A A Kirkwood, A Dey, D I Marks, A K McMillan, T F Menne, L Micklewright, P Patrick, S Purnell, C J Rowntree, P Smith, A K Fielding

**Affiliations:** 1 Barts Cancer Institute, The London School of Medicine, Queen Mary University of London, London, UK; 2 CR UK and UCL Cancer Trials Centre, London, UK; 3 Cancer Institute, University College London, London, UK; 4 Centre for Clinical Haematology, Nottingham City Hospital, Nottingham, UK; 5 United Bristol Healthcare Trust, Bristol, UK; 6 Newcastle Upon Tyne Hospitals NHS Foundation Trust, Newcastle Upon Tyne, UK; 7 Cardiff and Vale UHB, London, UK

## Abstract

Safety and efficacy data on pegylated asparaginase (PEG-ASP) in adult acute lymphoblastic leukaemia (ALL) induction regimens are limited. The UK National Cancer Research Institute UKALL14 trial NCT01085617 prospectively evaluated the tolerability of 1000 IU/m^2^ PEG-ASP administered on days 4 and 18 as part of a five-drug induction regimen in adults aged 25–65 years with *de novo* ALL. Median age was 46.5 years. Sixteen of the 90 patients (median age 56 years) suffered treatment-related mortality during initial induction therapy. Eight of the 16 died of sepsis in combination with hepatotoxicity. Age and Philadelphia (Ph) status were independent variables predicting induction death >40 versus ⩽40 years, odds ratio (OR) 18.5 (2.02–169.0), *P*=0.01; Ph− versus Ph+ disease, OR 13.60 (3.52–52.36), *P*<0.001. Of the 74 patients who did not die, 37 (50.0%) experienced at least one grade 3/4 PEG-ASP-related adverse event, most commonly hepatotoxicity (36.5%, *n*=27). A single dose of PEG-ASP achieved trough therapeutic enzyme levels in 42/49 (86%) of the patients tested. Although PEG-ASP delivered prolonged asparaginase activity in adults, it was difficult to administer safely as part of the UKALL14 intensive multiagent regimen to those aged >40 years. It proved extremely toxic in patients with Ph+ ALL, possibly owing to interaction with imatinib.

## Introduction

Depletion of extracellular asparagine by parenteral administration of the enzyme L-asparaginase is a key component of most current therapeutic strategies in acute lymphoblastic leukaemia (ALL). In children, intensive L-asparaginase treatment, typically delivered by pegylated *Escherichia coli*-derived L-asparaginase (PEG-ASP), improves clinical outcome,^1, 2, 3, 4, 5, 6^ offering a longer half-life^7^ and a lower risk of antiasparaginase antibody formation. Safety and efficacy is less well established in older adults,^8, 9^ and toxicity can be substantial^10^—in a phase 2 trial, failure to deliver the intended doses was closely correlated with advancing age.^11^

UKALL14 (NCT01085617) is an on-going, multicentre, phase 3 study that addresses several questions in the treatment of newly diagnosed adult ALL. A major study aim is to evaluate the addition of two doses of 1000 IU/m^2^ PEG-ASP to the standard induction regimen that had been evaluated in our previous study, UKALL12,^12^ in which non-pegylated *E. coli* L-asparaginase was given at 10 000 IU daily on days 17–28 of phase 1 induction. The only other change to the ‘backbone' induction regimen between the two consecutive national trials was the addition of a steroid prephase and the substitution of pulsed dexamethasone for prednisolone. The aim of these changes was to make our regimen more compatible with a 'paediatric-inspired' intensive approach.

The overall end point of the trial is event-free survival. However, a specific end point of the PEG-ASP evaluation is toxicity related to PEG-ASP. Secondary end points include rate of complete remission (CR), overall survival, minimal residual disease (MRD) quantitation at the end of the first phase of induction and antiasparaginase antibody formation. Here we report on the outcome of PEG-ASP administered during induction in the first consecutive 91 trial subjects. At this point, it was judged by the trial management group that a toxicity end point had been reached and a change was made to the PEG-ASP trial therapy.

## MATERIALS AND Methods

### UKALL14 induction phase 1 treatment

Eligible patients were aged ⩾25 and ⩽65 years with newly diagnosed ALL, irrespective of Philadelphia (Ph) chromosome status. There was no exclusion for poor organ function or performance status at diagnosis. Ethical approval was obtained from the UK National Research Ethics Committee. All patients gave written, informed consent, according to the Declaration of Helsinki. Patients received a 5–7-day prephase of dexamethasone 6 mg/m^2^/day followed by two sequential courses of induction therapy, termed induction phase 1 and induction phase 2, respectively. Patients with precursor B lineage ALL were randomised to receive chemotherapy alone or chemotherapy plus four doses of rituximab given on day (D) 3, D10, D17 and D24, PEG-ASP 1000 IU/m^2^ on D4 and D18, daunorubicin 60 mg/m^2^ and vincristine 1.4 mg/m^2^ (2 mg max.) on D1, D8, D15 and D21, dexamethasone 10 mg/m^2^ D1, −D4, D8–D11 and D15–D18 and a single 12.5 mg intrathecal methotrexate dose on D14. Patients with Ph chromosome-positive (Ph+) disease received continuous oral imatinib from D1, starting at 400 mg and escalating to 600 mg given daily throughout induction. Antibacterial, antiviral and antifungal prophylaxis was mandated, but centres used local policy for choice of agents. Granulocyte colony-stimulating factor support was strongly recommended. Routine antithrombotic prophylaxis was suggested but not mandated for all patients with platelet counts >50 × 10^9^/l. Anticoagulation with antithrombin replacement was recommended in the case of thrombosis. Routine coagulation factor replacement for laboratory-detected coagulopathy was specifically discouraged.

All patients had an initial assessment of response—including MRD response by *BCR-ABL* transcript monitoring or clonal immunoglobulin (Ig)/T-cell receptor gene rearrangement quantification—at the end of induction phase 1, which did not affect treatment decision. Formal assessment of response to induction therapy (outside the scope of this report) was documented after phase 2 induction.

**Figure 1:**
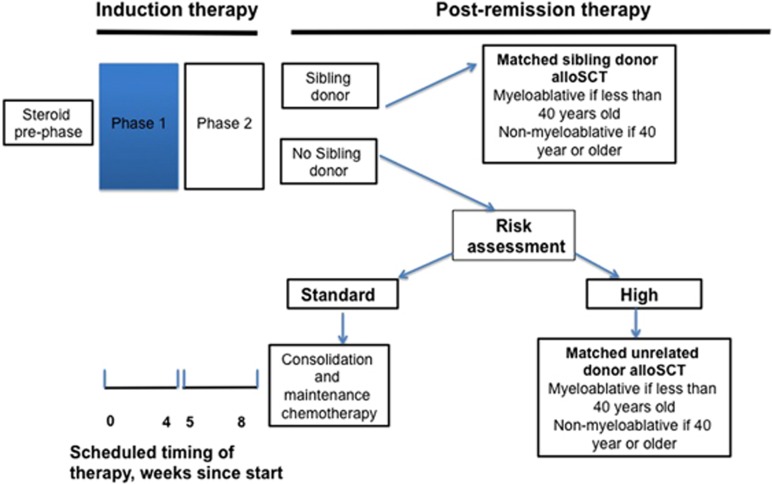
Schematic of UKALL14 treatment protocol. High risk features: karyotypes: t(9;22) t(4;11), low hypodiploidy near triploidy or complex, age >40 years, WBC ⩾30 × 10^9^/l (precursor B lineage ALL), ⩾100 × 10^9^/l (T-cell-ALL), molecular minimal residual disease positivity (>1 × 10^−4^) after induction phase 2.

A simplified schema of the remainder of UKALL14 treatment is provided in Figure 1.

### Causality assessment for toxicity and death

The standard Common Terminology Criteria for Adverse Events (CTCAE) reporting system (http://ctep.cancer.gov/protocolDevelopment/electronic_applications/ctc.htm) was used. Causality of events was attributed, as is standard practice, by both the local site Principal Investigator and the central study clinical team. Additional detailed questionnaires and Principal Investigator's narratives were received for all induction deaths allowing a more detailed analysis of individual events.

### PEG-ASP antibody assays

Antibodies against PEG-ASP (IgG and IgE) were measured by two indirect enzyme-linked immunosorbent assays, which detected anti-PEG-ASP and non-pegylated anti-*E. coli* (non-pegylated asparaginase). Seroconversion was reported with positivity in at least one assay with a clearly negative predose sample. Anti-asparaginase antibody ratio over negative control >1.1 was used to define positivity.

### Serum asparaginase activity by MAAT testing

PEG-ASP enzyme activity was quantified in sera using the MAAT^7^ assay. Therapeutic enzyme levels were defined as >100 IU/l.

### Statistical analysis

Induction phase 1 treatment-related death was defined as any death occurring before the start of phase 2 induction where the cause of death was not primarily attributable to progressive ALL. Logistic regression was used to examine risk potential factors for induction death and grade 3/4 adverse events (AEs). Factors with a conservative *P*<0.2 in the univariate analysis were included in the multivariable analyses. All analyses were conducted using Stata 14.0 (Stata Corporation, College Station, TX, USA). Non-fatal grade III–IV AEs causally related to PEG-ASP were those classified as probably or definitely related. All AEs were graded according to CTCAE version 4.0.

## Results

### Patient characteristics

Ninety-one eligible patients (from 37 centres) were enrolled onto UKALL14 between 30 December 2010 and 19 April 2012. One patient died before starting any treatment and has been excluded from all analyses. Patient characteristics at diagnosis are summarised in Table 1. The majority (59 of 90, 66%) of patients were aged >40 years and nearly a third (26/90, 29%) had Ph+ ALL. Most patients (92%) had performance scores of 0–1 at diagnosis. Median follow-up was 36.0 months (12 days–50.4 months).

### Induction deaths

**Figure 2:**
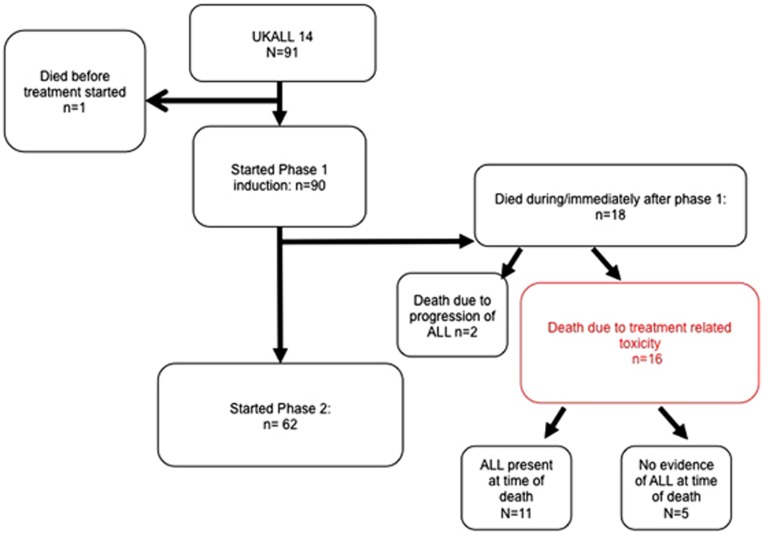
Flow chart of progress of the 91 patients enrolled. Grade 4 sepsis *n*=4, grade IV organ toxicity *n*=4 (hepatotoxicity *n*=1, pancreatitis *n*=1, hepatotoxicity plus neurological event *n*=1, hepatotoxicity plus thromboembolism together with sepsis *n*=1 and wrong diagnosis *n*=1, withdrawal of consent, *n*=1.

Progress through the induction therapy blocks is shown in Figure 2. Among those commencing phase 1 induction therapy (*n*=90), there were 18 early deaths. Two patients died of progressive ALL and the other 16 (16/90, 18%) deaths were related to induction treatment, occurring at a median time from start to induction of 23 days (range 10–53).

The causes of induction deaths are summarised in Table 2. In 12 of the 16 (75%), the causes of death were most often multifactorial; sepsis together with hepatotoxicity occurred in 8 of 16, 50.0%. Neutropenic sepsis alone occurred in 3 of the 16 patients, (18.8%). A causative organism was identified in 11 of the cases of sepsis, with a Gram-negative bacterial infection being responsible in 8 of those. Additional causes of death were: hepatotoxicity plus bowel ischaemia (*n*=2), acute coronary syndrome plus neutropenic sepsis (*n*=1), hepatotoxicity plus pancreatitis (*n*=1), and pulmonary haemorrhage (*n*=1). Nine of the 11 hepatotoxicity-related induction deaths were associated with grade 3–4 hyperbilirubinaemia. In total, half of the induction deaths were accompanied by recognised PEG-ASP toxicities (namely, those already listed in the Summary of Product Characteristics as occurring in 1:⩾1000 patients, or less common but clearly recognised as being related to PEG-ASP). There was no obvious association between baseline comorbidities and induction death.

All patients received D4 PEG-ASP. Sixty-four, including 5 of the 16 who suffered induction deaths, received the second dose on D18. For patients who died during induction, the median time from last dose of PEG-ASP to death was 13 days (range 6–50).

### Risk factors for induction death

Table 3 shows a univariate analysis of factors predictive of induction death. Age, Ph positivity and high-risk cytogenetics were all risk factors. Patients aged over 40 years had a more than 10-fold increase in risk of death during induction with Ph+ disease conferring a more than 8-fold increase compared with Ph−disease (odds ratio (OR): 8.65 (2.61–28.71), *P*<0.001). There was no relationship to baseline albumin levels or body mass index (BMI). The data monitoring committee also confirmed that there was no relationship to rituximab randomisation arm. The multivariable analysis is also shown in Table 3. Although high-risk cytogenetics appeared to be associated with induction death, this was driven by the presence of t(9;22), so this factor alone was included in the multivariable analysis. Age and Ph status remained significantly associated with induction death; in patients who were Ph−, there were no deaths in the 21 patients aged ⩽40 years and 5 in the 43 patients aged >40 years. In patients with Ph+ ALL, 1 of the 10 patients aged ⩽40 years died compared with 10 of the 16 who were aged >40 years.

### AEs during phase 1 induction therapy

Including the patients discussed above who subsequently died, 87 of the 91 patients (97%) experienced a grade 3–5 AE during induction phase 1; among these 46 (51%) suffered one or more recognised PEG-ASP toxicities; 34 had grade 3–5 AEs indicating liver dysfunction, including 22 with raised bilirubin. Other PEG-ASP toxicities included pancreatitis (*n*=3), intracranial haemorrhage (*n*=1), allergic reaction (*n*=3), coagulation disorder (*n*=4) and vascular events (*n*=6). Thirty-seven of the 74 surviving patients (50.0%) experienced at least one recognised grade 3–4 PEG-ASP-related, non-fatal toxicity summarised in Table 4. Hepatotoxicity—including biochemical markers of liver dysfunction—was the most frequent PEG-ASP-related toxicity (36.5%, *n*=27). Venous thromboembolism (4.1%, *n*=3), allergic reaction (4.1%, *n*=3) and pancreatitis (2.7%, *n*=2) were all reported but were relatively uncommon. A complete line-listing of all AE/serious AE as well as the subset known to be recognised toxicities of PEG-ASP from which Table 4 is derived is given in Supplementary Supplementary Table S1. As liver toxicity was so prominent and can be a key determinant of subsequent on-time therapy delivery, an analysis of any pretreatment factors associated with grade 3–4 hepatotoxicity was carried out. This is shown in Table 5. Older age and BMI were the only factors that showed a significant association (OR: 2.88 (1.62–15.44), *P*=0.005, for patients aged >40 years compared with those ⩽40 years and OR: 1.58 (1.02–2.44), *P*=0.041, for a 5 unit increase in BMI).

### Asparaginase activity, antiasparaginase antibody formation and correlation with MRD response

A trough level of asparaginase activity by MAAT testing^7, 13^ was assessed 14 days after the first (D4) PEG-ASP dose in 49 patients in whom serum was available (*n*=49). Therapeutic activity (enzyme level >100IU/l) was achieved in 42 of 49 (86%). The median enzyme level in those achieving therapeutic enzyme levels was 234 IU/l (range 101.5–602.8) as compared with 44.8IU/l (0–97.5) in those with subtherapeutic levels. There was no evidence of an association between age and achievement of therapeutic enzyme level. Molecular MRD at the end of phase 1 induction was documented in 27 patients. Molecular remission rates did not significantly differ between those achieving therapeutic enzyme levels of PEG-ASP and those who did not (14/26 compared with 3/4), suggesting that an early complete molecular response in this setting is not contingent upon therapeutic asparaginase enzyme activity. Anti-PEG-ASP antibody formation at D18 was assessed in 59 patients, and at that time point, there were no instances of seroconversion.

## Discussion

Successful achievement of CR during induction therapy for ALL is an absolute prerequisite for long-term disease-free survival. Numerous previous studies have suggested that induction regimens typically used in children give a better outcome for teenagers and younger adults than typical ‘adult' regimens.^14, 15, 16, 17, 18^ As a consequence, there is general acceptance of testing the outcome of increasing the intensity of non-myelosuppressive agents such as PEG-ASP in regimens used for older adults.

Within the first 91 patients enrolled, we noted significantly greater induction mortality in UKALL14 than in our previous trial, UKALL12. Induction death occurred in 16 of the first 90 (17.8%) treated patients, and 15 of the first 59 (25%) participants being aged >40 years (10/29 (33%) being aged >55 years). The median age of the patient population enrolled in this period was 46.5 years, with 29% of patients being aged >55 years. By comparison, the induction death rate in our previous trial, UKALL12/ECOG2993, was 6% overall (ages 15–60 years, median age 36 years)^12^ and 15% in the 55–65-year range (median age 56 years).^19^ UKALL14 did not exclude any patient with poor organ function or comorbidity at diagnosis, but we did not find any evidence of these factors having a causal contribution to PEG-ASP-related mortality.

Analysis of the cause of each death in UKALL14 included a complete written narrative from the treating physician as well as the standard case report forms for serious AE collection. Bacterial sepsis was revealed as a major contributor to the deaths. However, concomitant grade 3–4 PEG-ASP toxicities were very common among those suffering induction death, such that a recognised PEG-ASP toxicity was associated with half of the 16 induction deaths. It is impossible to disentangle the role of the investigational medicinal product (IMP) PEG-ASP from that of concomitant non-IMP induction agents. However, the introduction of dexamethasone instead of prednisolone was the only change that had been made to the standard backbone regimen between UKALL12 and UKALL14.

It is noteworthy that most of the induction deaths reported here occurred after an early, single (D4) dose of PEG-ASP, any toxicity of which would overlap with the myelosuppressive toxicity of anthracycline administered on D1, D8, D15 and D22. The 60 mg/m^2^ dose of anthracycline given in the UKALL12 regimen was well tolerated, but asparaginase was not started until D17. The earlier addition of PEG-ASP-associated liver dysfunction to subsequent myelosuppression-related sepsis may have been responsible for the overlapping toxicity. The fact that sepsis was a major contributor to the mortality supports that contention.

To date, only two other studies reporting the use of PEG-ASP specifically in adults have been published, both of which report a lower dose and/or different schedule of anthracycline. A phase 2 trial CALGB 9511^(ref. 11)^ reported on 102 adults treated with PEG-ASP at 2000 U/m^2^ subcutaneously, capped at 3750 U/m^2^, starting on D5 of the five-drug induction regimen. Although the regimen was reported to be 'tolerable', bilirubin >51.3 μm (3 mg/dl) occurred in 54% of patients. CR rate was only 77% but induction deaths are not separated from induction failures within the report. There was a statistically significant difference in median age of those achieving full dose delivery and asparagine depletion—32 years versus 48 years. In a more recent study from Douer *et al.*^20^ in 51 adults median age 32 years, upper age limit 57 years reported no deaths directly as a result of PEG-ASP in regimen, which included 2000 IU/m^2^ PEG-ASP given on D15. However, 3 of the 51 (6%) patients died of neutropenic sepsis during consolidation. In this case, daunorubicin was given at 60 mg/m^2^ intravenously on D1–D3. Grade 3–4 hyperbilirubinaemia (1/3 patients) or transaminitis (2/3 of patients) was very common. Douer *et al.*^20^ also reported long median intervals from PEG-ASP to bilirubin recovery, which affected subsequent chemotherapy delivery. The younger median age of patients in that study, the small number receiving concurrent imatinib (*N*=5), a more modest anthracycline dose and different scheduling of PEG-ASP administration so as not to coincide with the maximal myelosuppression may all have contributed to the difference in toxicity.

The German Multicentre Adult ALL Group have reported in abstract form^21^ on 1000 adult patients aged 15–55 years, treated on the German 07/2003 study in which a single dose of 1000–2000 U/m^2^ PEG-ASP was administered on D3 during induction with a reported rate of induction death rate of <5% and a grade IV hyperbilirubinaemia incidence of 16%. As with the study by Douer *et al.*,^20^ the younger median age (35 years) of the cohort in addition to the lower dose of anthracycline (45 versus the 60 mg/m^2^ used in the current study) may underpin the differences in toxicity.

It should be noted that the French GRAAALL group also reported that advancing age resulted in a higher cumulative incidence of chemotherapy-related deaths (23% versus 5%, respectively; *P*<0.001) and deaths in first CR (22% versus 5%, respectively; *P*<0.001) during 'paediatric-style' therapy, even when non-pegylated asparaginsae was used. In the study reported by Huguet *et al.*,^22^ ⩾45 years of age was the strongest predictor of severe toxicity,

As a consequence of the toxicity reported here, the UKALL14 protocol was amended to omit D4 PEG-ASP for those aged >40 years. Furthermore, PEG-ASP was completely removed from induction treatment for all patients with Ph+ ALL. In addition, owing to the high level of sepsis related to profound myelosuppression, the daunorubicin dose was halved to 30 mg/m^2^ on D1, D8, D15 and D22. One year following this amendment, when an additional 302 further patients had been recruited with 244 patients assessable for induction death, a repeat analysis showed only 6 (2.5%) phase 1 induction deaths (data not shown).

Univariate analysis demonstrated that older age and poor-risk cytogenetics—including t(9;22)—were significantly associated with induction death. On multivariable analysis, age and Ph positivity remained as independent risk factors for induction death, suggesting that the concomitant imatinib therapy only received by those with Ph+ ALL may have compounded the potential for PEG-ASP toxicity. The precise mechanism by which this might occur is not known. However, from the Summary of Product Characteristics for imatinib, ‘increased hepatic enzymes' is reported as common (1:10 to 1:100), hyperbilirubinaemia occurs in between 1:100 and 1:1000 patients and, finally, hepatic failure is noted as ‘rare', namely, occurring between 1:1000 and 1:10 000 cases. Hence, the potential for overlapping hepatotoxicity of PEG-ASP and imatinib is clearly present.

Half of the patients who did not experience an induction death experienced one or more grade 3–4 PEG-ASP toxicities from which they eventually recovered—most commonly, hepatotoxicity. Twenty-three percent of patients in this study experienced grade ⩾3 hyperbilirubinaemia; by definition, a limitation or exclusion to further dosing with many commonly used ALL therapeutics. On analysis of treatment delays in our previous trial, UKALL12/E2993 study showed that delays of >4 weeks were associated with poorer overall survival and event-free survival in patients undergoing allogeneic hematopoietic stem cell transplantation but not in patients undergoing postremission chemotherapy.^22^ Any long-term impact of any delays engendered by toxicity during initial induction therapy in this study will become clear when the trial is completed and we can evaluate any delays in relation to our primary end point, event-free survival.

Our pharmacokinetic studies indicate that a single dose of 1000 IU/m^2^ PEG-ASP generates therapeutic enzyme levels in most patients—86% of assessable patients had therapeutic levels of PEG-ASP activity 14 days after administration. A recent longitudinal analysis of PEG-ASP pharmacokinetics during a ‘paediatric' regimen in adult ALL demonstrated therapeutic enzyme activity in a similar proportion of adult patients for up to 21 days.^20^ In our study, antiasparaginase antibodies were not seen at the early time point documented.

In conclusion, we have shown that PEG-ASP achieves very effective asparagine depletion without antiasparaginase antibody formation in the majority of adult patients. However, toxicity can be substantial in older patients, making a 'paediatric-inspired' regimen of the type commonly used used in paediatric and young adult therapy difficult to deliver safely to those aged >40 years. Avoidance of overlapping toxicities and careful timing of administration will be of key importance to the more widespread use of this type of regimen in older adults.

As remission can be induced with minimal mortality in patients with Ph+ ALL,^23, 24^ we suggest that PEG-ASP is never coadministered with imatinib during induction therapy.

## Figures and Tables

**Table 1:** Patient characteristics at diagnosis

*Characteristic*	N *(%)*
*Lineage*	
B-precursor	77 (86)
T-cell	13 (14)
	
*Sex*	
Male	48 (53)
Female	42 (47)
	
*Age at entry (years)*	
Median (range)	46.50 (25–65)
⩾55	29 (32)
⩾41	59 (66)
	
*Presenting WBC ( × 10*^*9*^*/l)*	
Median (range)	9.26 (0.52–297.4)
<30	62 (69)
30–99.9	15 (17)
100+	13 (14)
	
*Cytogenetic risk status*	
High risk^a^	28 (31)
Low risk	42 (47)
Unknown^b^	20 (22)
	
*t(9;22)*	
Absent	64 (71)
Present	26 (29)
	
*Low hypodiploidy/near triploidy*	
Absent	65 (72)
Present	4 (4)
Failed/missing	21 (23)
	
*t(4;11)*	
Absent	75 (83)
Present	7 ( 8)
Failed/missing	8 (9)
	
*Complex karyotype*	
Absent	64 (71)
Present	5 (6)
Failed/missing	21 (23)
	
*Performance status*	
0	55 (61)
1	28 (31)
2	5 ( 6)
3	1 (1)
Missing	1 (1)
	
*BMI*	
Normal/underweight^c^	31 (34)
Overweight	25 (28)
Obese	34 (38)

Abbreviations: BMI, body mass index; WBC, white blood cell.

a High risk: t(9;22), t(4;11) low hypodiploidy/near triploidy or complex karyotypes.

b No high-risk factors and at least one risk factor is missing or failed.

c 30 in the normal range and 1 patient with a BMI of 17.

**Table 2:** Summary of deaths during induction

*ID*	*Patient details*	*Comorbidities*	*Hepatotoxicity (grade)*	*Neutropenic sepsis (organism)*	*Haemorrhage (site)*	*Thrombosis/visceral ischaemia (site)*	*Pancreatitis*	*Other*	*PEG-ASP doses*	*Days from last PEG-ASP dose*
1003	Age 54 years PH−	Liver cirrhosis	4	Not known					D4 and 18	19
1004	Age 46 years PH+	None		*Pseudomonas aeruginosa*		Acute coronary syndrome			D4	16
1011	Age 43 years PH+	None	4	*Escherichia coli*				Renal failure	D4 & 18	8
1021	Age 65 years PH−	None	4	*Pseudomonas aeruginosa*		Gastrointestinal			D4	50
1026	Age 64 years PH+	Depression	4	*Pseudomonas aeruginosa*					D4	20
1028	Age 38 years PH+	None		*Enterococcus faecium*					D4	15
1029	Age 60 years PH+	IHD	4	*Escherichia coli*					D4	14
1030	Age 49 years PH+	None			Pulmonary				D4	6
1039	Age 62 years PH+	None	2			Small bowel			D4	10
1043	Age 63 years PH−	Grade 4 renal failure	4	*Klebsiella pneumoniae*	Carotid artery puncture site				D4 and D18	43
1049	Age 56 years PH+	None	4	*Pseudomonas aeruginosa*					D4	11
1057	Age 57 years PH−	None	4			Small bowel			D4	20
1061	Age 54 years PH+	Paroxysmal atrial fibrillation	3				Yes	GI perforation	D4	10
1063	Age 62 years PH+	None		Coagulase-negative staph/*Enterococcus faecium*					D4 and D18	12
1068	Age 64 years PH+	None	4	Respiratory syncytial virus				Renal failure	D4	21
2007	Age 55 years PH−	None		*Pseudomonas aeruginosa*					D4 and D18	8

Abbreviations: IHD, ischaemic heart disease; PEG-ASP, pegylated asparaginase.

**Table 3:** Univariate and multivariate analysis of risk factors for induction death

*Risk factor*	*Events/*n	*Univariable*		*Multivariable*	
		*Odds ratio (95% CI)*	P*-value*	*Odds ratio (95% CI)*	P*-value*
*Age, years*					
⩽40	1/31	1.00	0.03	18.50 (2.02–169.0)	0.01
>40	15/59	10.23 (1.28–81.59)			
					
*Gender*					
Male	8/48	1.00	0.77	—	—
Female	8/42	1.18 (0.40–3.47)			
					
Baseline WBC per × 10^9^/l increment	16/90	1.00 (0.99–1.01)	0.56	—	
					
*Cytogenetics*^a^					
Standard risk	2/28	1.00	0.0029	—	—
High risk	13/42	5.83 (1.20–28.29)			
					
*Ph*					
Absent	5/64	1.00	<0.001	13.60 (3.53–52.36)	<0.001
Present	11/26	8.65 (2.61–28.71)			
					
Base albumin (g/l) (10 unit increment)	16/90	1.03 (0.40–2.64)	0.95	—	—
Base bilirubin (μmol/l) (10 unit increment)	16/90	0.91 (0.59–1.42)	0.69	—	—
BMI (5 unit increment)	16/90	1.29 (0.82–2.02)	0.27	—	—

Abbreviations: BMI, body mass index; CI, confidence interval; WBC, white blood cell.

a Analysed as no high-risk factors versus high-risk Ph and high-risk Ph+ gives HRs of 1.86 (0.24–14.64) and 9.53 (1.86–49.91), respectively.

**Table 4:** Patients who did not die during induction: summary of the grade 3/4 AEs recognised in SPC as being related to PEG-ASP^a^

*SOC/event term*	*Grade 3+ PEG-ASP known AEs,* N *(%)*
*Gastrointestinal disorders*	2 (3)
Pancreatitis	2 (3)
	
*Hepatobiliary disorders*	3 (4)
Liver failure	1 (1)
Liver dysfunction	2 (3)
	
*Immune system disorders*	3 (4)
Allergic reaction	3 (4)
	
*Investigations*	29 (39)
Lipase increased	1 (1)
Serum amylase increased	3 (4)
Alkaline phosphatase increased	15 (20)
Aspartate aminotransferase increased	4 (5)
Blood bilirubin increased	17 (23)
Alanine aminotransferase increased	10 (14)
GGT increased	5 (7)
	
*Metabolism and nutrition disorders*	3 (4)
Increased triglycerides	1 (1)
Hypoalbuminaemia	2 (3)
	
*Nervous system disorders*	1 (1)
Intracranial haemorrhage	1 (1)
	
*Vascular disorders*	4 (5)
Pulmonary embolism	1 (1)
Thromboembolic event	3 (4)
	
*Non-CTCAE terms*	
Coagulation disorder	3 (4)
	
Any liver event	27 (36)
Any toxicity	37 (50)

Abbreviations: AE, adverse event; CTCAE, Common Terminology Criteria; GGT, gamma glutamyl transpeptidase; PEG-ASP, pegylated asparaginase; SPC, Summary of Product Characteristics; SOC, System Organ Class.

a A complete line listing of grade 3+ AE/serious AE according to CTCAE criteria with assignment of causality by both site and by trial management group is provided in Supplementary Table S1.

**Table 5:** Analysis of risk factors for liver-related grade 3/4 adverse events during phase 1 induction in patients who did not have an induction death

*Risk factor*	*Grade 3/4 liver AEs*		
	*Events/*n	*Odds ratio (95% CI)*	P*-value*
*Age*			
⩽40	5/30	1.00	0.005
>40	22/44	2.9 (1.62–15.44)	
			
*Sex*			
Male	16/40	1.00	0.50
Female	11/34	0.72 (0.28–1.87)	
			
Baseline WBC per × 10^9^/l increment	27/74	1.00 (0.99–1.01)	0.71
			
*Cytogenetics*			
Standard risk	10/26	1.00	0.97
High risk	11/29	0.98 (0.33–2.91)	
			
*Ph*			
Absent	20/59	1.00	0.36
Present	7/15	1.71 (0.54–5.38)	
			
Base albumin (g/l) (10 unit increment)	27/74	0.89 (0.41–1.92)	0.77
Base bilirubin (μmol/l) (10 unit increment)	27/74	1.02 (0.81–1.30)	0.86
BMI (5 unit increment)	27/74	1.58 (1.02–2.44)	0.041

Abbreviations: AE, adverse event; BMI, body mass index; CI, confidence interval; WBC, white blood cell.
