# Supplementary material for: Pegylated-asparaginase during induction therapy for adult acute lymphoblastic leukaemia: toxicity data from the UKALL14 trial
Source: Leukemia. 2016 Sep 9;31(1):58–64. doi: 10.1038/leu.2016.219 (PMC5154375; doi:10.1038/leu.2016.219)
Supplement: Supplementary Information [file leu2016219x1.docx]

**Supplementary results**

**Supplementary table 1 Grade 3+ AEs/SAEs in induction 1 (All patients)**

| **SOC/event term** | | **All Grade 3+** | **Grade 3+ PEG-ASP related (site’s assessment)** | **Grade 3+ PEG-ASP related (TMG’s assessment)** |
| --- | --- | --- | --- | --- |
|  |  | N(%) | N(%) | N(%) |
| **Blood And Lymphatic System Disorders** | | **72 (80.00)** | **4 (4.44)** | **0** |
|  | Anaemia | 63 (70.00) | 2 (2.22) | 0 |
|  | Febrile Neutropenia | 41 (45.56) | 1 (1.11) | 0 |
|  | Disseminated Intravascular Coagulation | 3 (3.33) | 1 (1.11) | 0 |
| **Gastrointestinal Disorders** | | **21 (23.33)** | **3 (3.33)** | **3 (3.33)** |
|  | Vomiting | 1 (1.11) | 0 | 0 |
|  | Colonic Perforation | 2 (2.22) | 0 | 0 |
|  | Nausea | 4 (4.44) | 0 | 0 |
|  | Neutropenic Typhlitis | 1 (1.11) | 0 | 0 |
|  | Abdominal Pain | 2 (2.22) | 0 | 0 |
|  | Sore Mouth/Throat | 1 (1.11) | 0 | 0 |
|  | Typhlitis | 2 (2.22) | 0 | 0 |
|  | Sore Mouth | 1 (1.11) | 1 (1.11) | 0 |
|  | Diarrhoea | 6 (6.67) | 0 | 0 |
|  | Mouth Sores | 1 (1.11) | 0 | 0 |
|  | Mucocitis | 2 (2.22) | 0 | 0 |
|  | Toothache | 1 (1.11) | 0 | 0 |
|  | Enterocolitis | 1 (1.11) | 0 | 0 |
|  | Pancreatitis | 3 (3.33) | 2 (2.22) | 3 (3.33) |
| **General Disorders And Administration Site Conditions** | | **18 (20.00)** | **3 (3.33)** | **0** |
|  | Multi-Organ Failure | 3 (3.33) | 1 (1.11) | 0 |
|  | Fatigue | 4 (4.44) | 1 (1.11) | 0 |
|  | Fever | 3 (3.33) | 0 | 0 |
|  | Pain | 13 (14.44) | 1 (1.11) | 0 |
| **Hepatobiliary Disorders** | | **3 (3.33)** | **3 (3.33)** | **3 (3.33)** |
|  | Liver Failure | 1 (1.11) | 1 (1.11) | 1 (1.11) |
|  | Liver Dysfunction | 2 (2.22) | 2 (2.22) | 2 (2.22) |
| **Immune System Disorders** | | **3 (3.33)** | **1 (1.11)** | **3 (3.33)** |
|  | Allergic Reaction | 3 (3.33) | 1 (1.11) | 3 (3.33) |
| **Infections And Infestations** | | **57 (63.33)** | **2 (2.22)** | **0** |
|  | Infection - Viral | 6 (6.67) | 0 | 0 |
|  | Upper Respiratory Infection | 1 (1.11) | 0 | 0 |
|  | Lung Infection | 4 (4.44) | 0 | 0 |
|  | Infection - Unknown Aetiology | 11 (12.22) | 0 | 0 |
|  | Sepsis | 32 (35.56) | 1 (1.11) | 0 |
|  | Prostatitis | 1 (1.11) | 0 | 0 |
|  | Infection - Fungal | 9 (10.00) | 1 (1.11) | 0 |
|  | Device Related Infection | 2 (2.22) | 0 | 0 |
|  | Infection (Bacterial) | 34 (37.78) | 0 | 0 |
|  | Pericardial Abscess | 1 (1.11) | 0 | 0 |
|  | Hepatic Infection | 1 (1.11) | 0 | 0 |
| **Injury, Poisoning And Procedural Complications** | | **1 (1.11)** | **0** | **0** |
|  | Unhealed Wounds | 1 (1.11) | 0 | 0 |
| **Investigations** | | **79 (87.78)** | **28 (31.11)** | **37 (41.11)** |
|  | White Blood Cell Decreased | 20 (22.22) | 1 (1.11) | 0 |
|  | Neutropenia | 48 (53.33) | 4 (4.44) | 0 |
|  | Lymphocyte Count Decreased | 1 (1.11) | 0 | 0 |
|  | Pancytopenia | 34 (37.78) | 0 | 0 |
|  | Neutrophil Count Decreased | 26 (28.89) | 1 (1.11) | 0 |
|  | Lipase Increased | 1 (1.11) | 0 | 1 (1.11) |
|  | Serum Amylase Increased | 4 (4.44) | 3 (3.33) | 4 (4.44) |
|  | Granulocytopenia | 25 (27.78) | 0 | 0 |
|  | Cpk Increased | 1 (1.11) | 0 | 0 |
|  | Platelet Count Decreased | 17 (18.89) | 0 | 0 |
|  | Crp Elevated | 1 (1.11) | 0 | 0 |
|  | Leucopenia | 37 (41.11) | 3 (3.33) | 0 |
|  | Myelosuppression | 21 (23.33) | 0 | 0 |
|  | Anuria | 1 (1.11) | 0 | 0 |
|  | Alkaline Phosphatase Increased | 16 (17.78) | 13 (14.44) | 16 (17.78) |
|  | Aspartate Aminotransferase Increased | 4 (4.44) | 2 (2.22) | 4 (4.44) |
|  | Blood Bilirubin Increased | 22 (24.44) | 14 (15.56) | 22 (24.44) |
|  | Abnormal Lfts | 1 (1.11) | 0 | 1 (1.11) |
|  | Urea Increased | 1 (1.11) | 0 | 0 |
|  | Alanine Aminotransferase Increased | 14 (15.56) | 7 (7.78) | 14 (15.56) |
|  | Aptt Increased | 1 (1.11) | 0 | 1 (1.11) |
|  | Ggt Increased | 6 (6.67) | 3 (3.33) | 6 (6.67) |
| **Metabolism And Nutrition Disorders** | | **20 (22.22)** | **3 (3.33)** | **4 (4.44)** |
|  | Increased Triglycerides | 1 (1.11) | 1 (1.11) | 1 (1.11) |
|  | Tumour Lysis Syndrome | 2 (2.22) | 0 | 0 |
|  | Dehydration | 2 (2.22) | 0 | 0 |
|  | Anorexia | 7 (7.78) | 0 | 0 |
|  | Hypophosphatemia | 2 (2.22) | 0 | 0 |
|  | Hypocalcaemia | 1 (1.11) | 0 | 0 |
|  | Hyperglycaemia | 5 (5.56) | 0 | 0 |
|  | Hyponatraemia | 3 (3.33) | 0 | 0 |
|  | Hyperuricemia | 1 (1.11) | 0 | 0 |
|  | Metabolic Acidosis | 1 (1.11) | 1 (1.11) | 0 |
|  | Hypokalaemia | 1 (1.11) | 0 | 0 |
|  | Glucose Intolerance | 1 (1.11) | 0 | 0 |
|  | Hypalbuminaemia | 3 (3.33) | 1 (1.11) | 3 (3.33) |
| **Nervous System Disorders** | | **13 (14.44)** | **0** | **1 (1.11)** |
|  | Headache | 3 (3.33) | 0 | 0 |
|  | Seizures | 1 (1.11) | 0 | 0 |
|  | Vasovagal Attack | 1 (1.11) | 0 | 0 |
|  | Loss Of Consciousness | 7 (7.78) | 0 | 0 |
|  | Intracranial Haemorrhage | 1 (1.11) | 0 | 1 (1.11) |
|  | Encephalopathy | 1 (1.11) | 0 | 0 |
|  | Depressed Level Of Consciousness | 1 (1.11) | 0 | 0 |
|  | Syncope | 1 (1.11) | 0 | 0 |
| **Psychiatric Disorders** | | **4 (4.44)** | **0** | **0** |
|  | Depression | 2 (2.22) | 0 | 0 |
|  | Agitation | 1 (1.11) | 0 | 0 |
|  | Anxiety | 1 (1.11) | 0 | 0 |
| **Renal And Urinary Disorders** | | **2 (2.22)** | **0** | **0** |
|  | Hydronephrosis | 1 (1.11) | 0 | 0 |
|  | Renal Failure | 1 (1.11) | 0 | 0 |
| **Respiratory, Thoracic And Mediastinal Disorders** | | **5 (5.56)** | **0** | **0** |
|  | Dyspnoea | 3 (3.33) | 0 | 0 |
|  | Respiratory Failure | 1 (1.11) | 0 | 0 |
|  | Pleural Effusion | 1 (1.11) | 0 | 0 |
|  | Hypoxia | 1 (1.11) | 0 | 0 |
| **Skin And Subcutaneous Tissue Disorders** | | **2 (2.22)** | **0** | **0** |
|  | Rash | 2 (2.22) | 0 | 0 |
| **Surgical And Medical Procedures** | | **1 (1.11)** | **0** | **1 (1.11)** |
|  | Haemorrhage Post Central Line Insertion | 1 (1.11) | 0 | 1 (1.11) |
| **Vascular Disorders** | | **12 (13.33)** | **6 (6.67)** | **6 (6.67)** |
|  | Pulmonary Embolism | 1 (1.11) | 0 | 1 (1.11) |
|  | Visceral Arterial Ischaemia | 1 (1.11) | 1 (1.11) | 1 (1.11) |
|  | Bowel Ischaemia | 2 (2.22) | 1 (1.11) | 2 (2.22) |
|  | Thromboembolic Event | 4 (4.44) | 3 (3.33) | 4 (4.44) |
|  | Hypotension | 6 (6.67) | 1 (1.11) | 0 |
|  |  |  |  |  |
| **Non CTC AE terms:** | |  |  |  |
|  | Coagulation Disorder | 4 (4.44) | 2 (2.22) | 4 (4.44) |
|  | Fluid Overload | 1 (1.11) | 0 | 0 |
|  | Oedema | 1 (1.11) | 0 | 0 |
|  |  |  |  |  |
|  | Peripheral Oedema | 1 (1.11) | 0 | 0 |
|  |  |  |  |  |
| **Any Toxicity** | | **87 (96.67)** | **37 (41.11)** | **46 (51.11)** |

**Supplementary table 1 Grade 3+ AEs/SAEs in induction 1 (Non induction deaths only):**

| **SOC/event** | | **All Grade 3+** | **Grade 3+ PEG-ASP related (site’s assessment)** | **Grade 3+ PEG-ASP related (TMG’s assessment)** |
| --- | --- | --- | --- | --- |
|  |  | N(%)  **60 (81.08)** | N(%)  **3 (4.05)** | N(%)  **0** |
| **Blood And Lymphatic System Disorders** | |  |  |  |
|  | Disseminated Intravascular Coagulation | 2 (2.70) | 1 (1.35) | 0 |
|  | Anaemia | 52 (70.27) | 2 (2.70) | 0 |
|  | Febrile Neutropenia | 34 (45.95) | 0 | 0 |
| **Gastrointestinal Disorders** | | **17 (22.97)** | **3 (4.05)** | **2 (2.70)** |
|  | Typhlitis | 2 (2.70) | 0 | 0 |
|  | Vomiting | 1 (1.35) | 0 | 0 |
|  | Sore Mouth/Throat | 1 (1.35) | 0 | 0 |
|  | Neutropenic Typhlitis | 1 (1.35) | 0 | 0 |
|  | Nausea | 3 (4.05) | 0 | 0 |
|  | Abdominal Pain | 2 (2.70) | 0 | 0 |
|  | Mucocitis | 2 (2.70) | 0 | 0 |
|  | Diarrhoea | 6 (8.11) | 0 | 0 |
|  | Mouth Sores | 1 (1.35) | 0 | 0 |
|  | Sore Mouth | 1 (1.35) | 1 (1.35) | 0 |
|  | Toothache | 1 (1.35) | 0 | 0 |
|  | Pancreatitis | 2 (2.70) | 2 (2.70) | 2 (2.70) |
| **General Disorders And Administration Site Conditions** | | **10 (13.51)** | **1 (1.35)** | **0** |
|  | Fever | 2 (2.70) | 0 | 0 |
|  | Multi-Organ Failure | 1 (1.35) | 0 | 0 |
|  | Fatigue | 3 (4.05) | 1 (1.35) | 0 |
|  | Pain | 7 (9.46) | 0 | 0 |
| **Hepatobiliary Disorders** | | **3 (4.05)** | **3 (4.05)** | **3 (4.05)** |
|  | Liver Failure | 1 (1.35) | 1 (1.35) | 1 (1.35) |
|  | Liver Dysfunction | 2 (2.70) | 2 (2.70) | 2 (2.70) |
| **Immune System Disorders** | | **3 (4.05)** | **1 (1.35)** | **3 (4.05)** |
|  | Allergic Reaction | 3 (4.05) | 1 (1.35) | 3 (4.05) |
| **Infections And Infestations** | | **45 (60.81)** | **1 (1.35)** | **0** |
|  | Infection - Viral | 4 (5.41) | 0 | 0 |
|  | Prostatitis | 1 (1.35) | 0 | 0 |
|  | Hepatic Infection | 1 (1.35) | 0 | 0 |
|  | Lung Infection | 3 (4.05) | 0 | 0 |
|  | Sepsis | 21 (28.38) | 0 | 0 |
|  | Infection - Unknown Aetiology | 11 (14.86) | 0 | 0 |
|  | Pericardial Abscess | 1 (1.35) | 0 | 0 |
|  | Infection (Bacterial) | 28 (37.84) | 0 | 0 |
|  | Upper Respiratory Infection | 1 (1.35) | 0 | 0 |
|  | Device Related Infection | 1 (1.35) | 0 | 0 |
|  | Infection - Fungal | 9 (12.16) | 1 (1.35) | 0 |
| **Investigations** | | **65 (87.84)** | **24 (32.43)** | **29 (39.19)** |
|  | Aspartate Aminotransferase Increased | 4 (5.41) | 2 (2.70) | 4 (5.41) |
|  | Lipase Increased | 1 (1.35) | 0 | 1 (1.35) |
|  | Leucopenia | 33 (44.59) | 2 (2.70) | 0 |
|  | White Blood Cell Decreased | 11 (14.86) | 1 (1.35) | 0 |
|  | CPK Increased | 1 (1.35) | 0 | 0 |
|  | Granulocytopenia | 20 (27.03) | 0 | 0 |
|  | Neutropenia | 43 (58.11) | 2 (2.70) | 0 |
|  | Myelosuppression | 15 (20.27) | 0 | 0 |
|  | Platelet Count Decreased | 13 (17.57) | 0 | 0 |
|  | Serum Amylase Increased | 3 (4.05) | 2 (2.70) | 3 (4.05) |
|  | Neutrophil Count Decreased | 18 (24.32) | 1 (1.35) | 0 |
|  | GGT Increased | 5 (6.76) | 3 (4.05) | 5 (6.76) |
|  | Pancytopenia | 27 (36.49) | 0 | 0 |
|  | Anuria | 1 (1.35) | 0 | 0 |
|  | Alanine Aminotransferase Increased | 10 (13.51) | 7 (9.46) | 10 (13.51) |
|  | Blood Bilirubin Increased | 17 (22.97) | 12 (16.22) | 17 (22.97) |
|  | Alkaline Phosphatase Increased | 15 (20.27) | 13 (17.57) | 15 (20.27) |
| **Metabolism And Nutrition Disorders** | | **17 (22.97)** | **3 (4.05)** | **3 (4.05)** |
|  | Hypokalaemia | 1 (1.35) | 0 | 0 |
|  | Hyperuricemia | 1 (1.35) | 0 | 0 |
|  | Dehydration | 2 (2.70) | 0 | 0 |
|  | Anorexia | 7 (9.46) | 0 | 0 |
|  | Tumour Lysis Syndrome | 1 (1.35) | 0 | 0 |
|  | Hypalbuminaemia | 2 (2.70) | 1 (1.35) | 2 (2.70) |
|  | Metabolic Acidosis | 1 (1.35) | 1 (1.35) | 0 |
|  | Hyponatraemia | 2 (2.70) | 0 | 0 |
|  | Increased Triglycerides | 1 (1.35) | 1 (1.35) | 1 (1.35) |
|  | Hyperglycaemia | 4 (5.41) | 0 | 0 |
|  | Hypocalcaemia | 1 (1.35) | 0 | 0 |
|  | Glucose Intolerance | 1 (1.35) | 0 | 0 |
| **Nervous System Disorders** | | **7 (9.46)** | **0** | **1 (1.35)** |
|  | Intracranial Haemorrhage | 1 (1.35) | 0 | 1 (1.35) |
|  | Depressed Level Of Consciousness | 1 (1.35) | 0 | 0 |
|  | Syncope | 1 (1.35) | 0 | 0 |
|  | Seizures | 1 (1.35) | 0 | 0 |
|  | Loss Of Consciousness | 2 (2.70) | 0 | 0 |
|  | Headache | 2 (2.70) | 0 | 0 |
|  | Vasovagal Attack | 1 (1.35) | 0 | 0 |
| **Psychiatric Disorders** | | **3 (4.05)** | **0** | **0** |
|  | Anxiety | 1 (1.35) | 0 | 0 |
|  | Depression | 2 (2.70) | 0 | 0 |
| **Renal And Urinary Disorders** | | **1 (1.35)** | **0** | **0** |
|  | Hydronephrosis | 1 (1.35) | 0 | 0 |
| **Respiratory, Thoracic And Mediastinal Disorders** | | **3 (4.05)** | **0** | **0** |
|  | Hypoxia | 1 (1.35) | 0 | 0 |
|  | Dyspnoea | 2 (2.70) | 0 | 0 |
|  | Respiratory Failure | 1 (1.35) | 0 | 0 |
| **Skin And Subcutaneous Tissue Disorders** | | **2 (2.70)** | **0** | **0** |
|  | Rash | 2 (2.70) | 0 | 0 |
| **Vascular Disorders** | | **7 (9.46)** | **4 (5.41)** | **4 (5.41)** |
|  | Thromboembolic Event | 3 (4.05) | 3 (4.05) | 3 (4.05) |
|  | Pulmonary Embolism | 1 (1.35) | 0 | 1 (1.35) |
|  | Hypotension | 3 (4.05) | 1 (1.35) | 0 |
|  |  |  |  |  |
| **Non CTC AAE** | |  |  |  |
|  | Coagulation Disorder | 3 (4.05) | 1 (1.35) | 3 (4.05) |
|  |  |  |  |  |
|  |  |  |  |  |
| **Any Toxicity** | | **71 (95.95)**  **29 (39.19)** | |  |
